# Glial-derived mitochondrial signals affect neuronal proteostasis and aging

**DOI:** 10.1126/sciadv.adi1411

**Published:** 2023-10-13

**Authors:** Raz Bar-Ziv, Naibedya Dutta, Adam Hruby, Edward Sukarto, Maxim Averbukh, Athena Alcala, Hope R. Henderson, Jenni Durieux, Sarah U. Tronnes, Qazi Ahmad, Theodore Bolas, Joel Perez, Julian G. Dishart, Matthew Vega, Gilberto Garcia, Ryo Higuchi-Sanabria, Andrew Dillin

**Affiliations:** ^1^ Department of Molecular and Cellular Biology, Howard Hughes Medical Institute, The University of California, Berkeley, Berkeley, CA 94720, USA.; ^2^ Leonard Davis School of Gerontology, University of Southern California, Los Angeles, CA 90089, USA.

## Abstract

The nervous system plays a critical role in maintaining whole-organism homeostasis; neurons experiencing mitochondrial stress can coordinate the induction of protective cellular pathways, such as the mitochondrial unfolded protein response (UPR^MT^), between tissues. However, these studies largely ignored nonneuronal cells of the nervous system. Here, we found that UPR^MT^ activation in four astrocyte-like glial cells in the nematode, *Caenorhabditis elegans*, can promote protein homeostasis by alleviating protein aggregation in neurons. Unexpectedly, we find that glial cells use small clear vesicles (SCVs) to signal to neurons, which then relay the signal to the periphery using dense-core vesicles (DCVs). This work underlines the importance of glia in establishing and regulating protein homeostasis within the nervous system, which can then affect neuron-mediated effects in organismal homeostasis and longevity.

## Teaser

Glial cells sense mitochondrial stress and signal a beneficial stress signal to promote neuronal health and longevity.

## INTRODUCTION

Aging is a complex and gradual process accompanied by a progressive decline in physiological integrity and function. Underlying this physiological decline is the loss of essential cellular functions, concomitant with the accumulation of cellular and molecular damage, such as the increase in DNA mutations and accumulation of misfolded proteins, causing a decline in organellar and cellular function (*1*, *2*). One of the key organ systems in aging is the nervous system. The nervous system not only is affected by aging, as exemplified by neurodegenerative diseases, but also can modulate the aging process of the entire organism (*3*). The link between the nervous system and aging is governed by its role as a central regulatory hub, mediating the maintenance of homeostasis in response to fluctuations in the external and internal environments, intracellularly and intercellularly, within and between different tissues. One level of homeostasis that neurons are tasked with maintaining and coordinating across tissues is the homeostatic state of protein folding (proteostasis and protein homeostasis) (*4*). Proteostasis is one of the key features declining with age, due to the reduced ability to counteract misfolding and aggregation of proteins, a hallmark of many neurodegenerative diseases, such as Huntington’s disease (HD) (*5*). Neurons are able to regulate protein homeostasis across tissues for several pathways that are dedicated to protecting different cellular compartments (*6*, *7*), such as the mitochondrion (*8*), with activation of the pathway prolonging life span, by coordinating mitochondrial protein homeostasis across tissues. This protective pathway, the unfolded protein response of mitochondria (UPR^MT^) (*9*), is triggered upon insults to the organelle and causes the activation of a coordinated transcriptional response, which includes the up-regulation of mitochondrially targeted chaperones and proteases, modulating cellular processes to alleviate the burden of stress on mitochondria. Neurons secrete molecules that allow coordination of the UPR^MT^ across tissues, using dense-core vesicles (DCVs), serotonin, and the WNT-like ligand EGL-20 to trigger a response in peripheral tissues, such as the intestine (*10*, *11*).

Historically, much scientific work interrogating the homeostatic roles of the nervous system focused on neurons. While it is clear that glia, the other main cell type of the nervous system, can serve many roles in neuronal development and function, these roles are normally associated with support roles, including regulating cell number, neuronal migration, axon specification and growth, synapse formation and pruning, ion homeostasis, and synaptic plasticity and providing metabolic support for neurons (*12*, *13*). However, in recent years, it has become increasingly clear that glial health can affect aging and progression of neurodegenerative diseases, like Alzheimer’s disease (AD) (*14*). For example, expression of apolipoprotein E4 (ApoE4), one of the strongest risk factors for AD, specifically in astrocytes resulted in increased neuronal tau aggregation (*15*). Moreover, hyperactivation of the unfolded protein response of the endoplasmic reticulum (UPR^ER^), which drives ER stress resilience, solely in astrocyte-like glial cells resulted in a significant life-span extension in *Caenorhabditis elegans* (*16*). While these studies show the importance of glial function in organismal health, what they lacked is an active function of glia in promoting these beneficial effects. To uncover an active role for glia in stress signaling and longevity, we aimed to determine whether glial cells can sense mitochondrial stress and initiate an organism-wide response to promote mitochondrial stress resilience and longevity. We used multiple genetic methods to activate UPR^MT^ in nonneuronal cells, including cell-type–specific application of mitochondrial stress and direct activation of the UPR^MT^ in the absence of stress. We found that, regardless of method, activation of UPR^MT^ in a small subset of glial cells, the cephalic sheath (CEPsh) glia, provided robust organismal benefits, including prolonged life span and increased resistance to oxidative stress. Perhaps most unique in this model is that UPR^MT^ activation in CEPsh glia promotes neuronal health by alleviating protein aggregation in neurons of an HD model. CEPsh glia directly communicate with neurons through the release of small clear vesicles (SCVs) and relay the coordination to the periphery via downstream neuronal mechanisms. This glia to neuron signal results in induction of the UPR^MT^ in distal tissues, through a cell nonautonomous mechanism, which is dependent on the canonical UPR^MT^ pathway, yet unexpectedly distinct from paradigms where UPR^MT^ is directly activated in neurons. Collectively, these results reveal a previously unknown function for CEPsh glia in sensing mitochondrial stress, which initiates a signal to promote protein homeostasis in neurons and ultimately prolongs longevity. Therefore, glial cells serve as one of the upstream mediators of mitochondrial stress and its coordination across the entire organism.

## RESULTS

### Activation of the UPR^MT^ in glia elicits beneficial effects for the entire organism

**Fig. 1.**
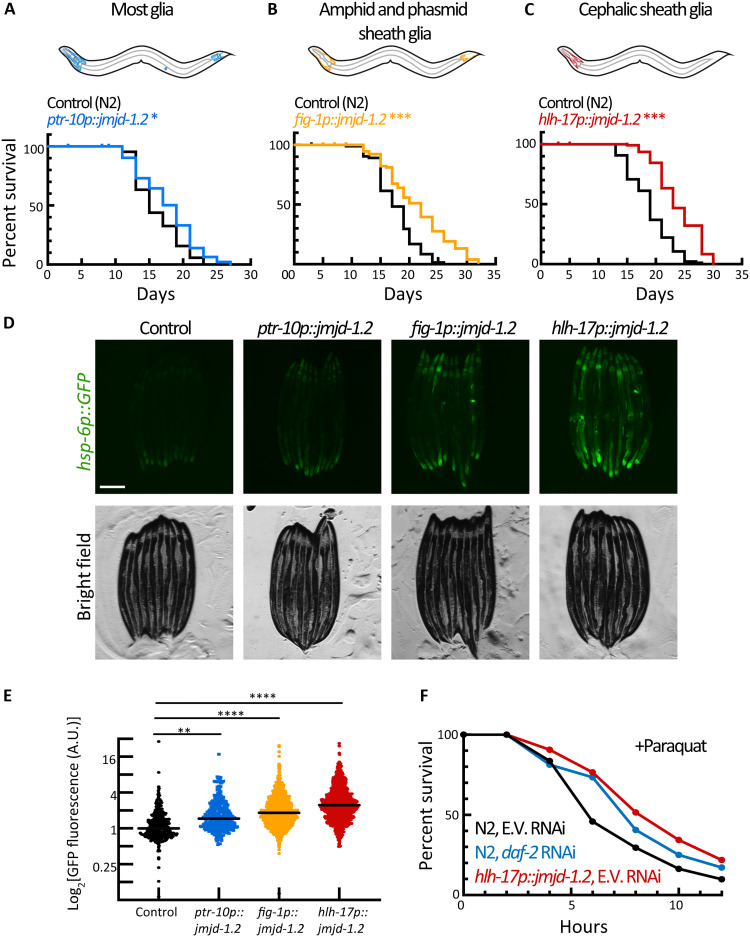
Glial activation of *jmjd-1.2* prolongs life span, improves stress resistance, and induces the cell nonautonomous UPR^MT^. (**A**) Survival of animals expressing *jmjd-1.2* in most glia (blue) compared to a control N2 population (black). *P = 0.0158. (**B**) Survival of animals expressing *jmjd-1.2* in amphid and phasmid sheath glia (orange) compared to a control N2 population. Log-rank test, ***P < 0.001. (**C**) Survival of animals expressing *jmjd-1.2* in the CEPsh glia (red) compared to a control N2 population. ***P < 0.001. (**D**) Representative fluorescent micrographs of UPR^MT^ reporter worms (*hsp-6p::GFP*) expressing *jmjd-1.2* under the indicated promoters. Scale bar, 250 μm. (**E**) Integrated fluorescence intensity is measured across an entire worm using a large-particle biosorter and normalized to size as described in Methods. Fold change was calculated normalized to a control (*hsp-6p::GFP*) population (*n* > 300 per group). One-way analysis of variance (ANOVA) with Tukey’s multiple comparisons test, **P < 0.01 and ****P < 0.0001. See also fig. S1G. A.U., arbitrary units. (**F**) Survival of animals expressing glial *jmjd-1.2* (red) under paraquat stress as compared to a control population (black). Day 1 adult animals were exposed to 100 mM paraquat in M9 solution and scored every 2 hours for survival. Worms fed with *daf-2* RNA interference (RNAi) were used as a positive control (green) (*n* = 60 per group); see also fig. S1H.

To activate the UPR^MT^ in glial cells, we leveraged the ability of JMJD-1.2 [JumonjiC (JmjC) domain–containing protein], a histone demethylase, to induce a robust UPR^MT^ in the absence of stress (*17*). Specifically, we overexpressed *jmjd-1.2* under several established glial promoters (*18*), targeting most glial cells, or glial subtypes using specific promoters. The activation of UPR^MT^ in most glial cells resulted in a mild life-span extension (Fig. 1A and fig. S1A). We found that overexpressing *jmjd-1.2* in either amphid sheath and phasmid sheath glia (*fig-1p*) or the four CEPsh glia (*hlh-17p*) alone was not only sufficient to prolong life-span but had a more profound effect than overexpressing *jmjd-1.2* in most glial cells simultaneously (Fig. 1, B and C). On the cellular level, the activation of the UPR^MT^ in all different glial subtypes was also able to trigger the activation of the UPR^MT^ in distal intestinal cells, as can be observed by the activation of a fluorescent reporter under the regulation of a mitochondrial chaperone promoter (*hsp-6p::GFP*) (Fig. 1, D and E). Similar to life-span extensions, overexpression of *jmjd-1.2* in CEPsh glia had the most profound effect on distal UPR^MT^ activation. We confirm that this is a beneficial and functional activation of UPR^MT^ as these animals also show increased resistance to paraquat (Fig. 1F and fig. S1G), a drug that increases superoxide levels mainly in the mitochondria (*19*). Animals with glial *jmjd-1.2* overexpression exhibit a similar increase in paraquat resistance to animals with *daf-2* knockdown, a condition with one of the highest recorded life-span extensions (*20*), primarily through oxidative stress resistance (*21*).

The most profound peripheral UPR^MT^ activation and life-span extension was observed when overexpressing *jmjd-1.2* in CEPsh glia, the four astrocyte-like cells that associate with sensory organs and extend processes that wrap around the nerve ring, the major neuropil of *C. elegans* (*12*). Therefore, we focused our efforts on this specific glial subtype (heretofore referred to as glial *jmjd-1.2*). To determine whether CEPsh glia can elicit nonautonomous signals in direct response to stress rather than solely via ectopic overexpression of UPR^MT^ activators, we induced mitochondrial stress specifically in CEPsh glia in two different ways. First, we use expression of a mitochondrially targeted KillerRed, which generates highly cytotoxic reactive oxygen species when fluorescently activated. KillerRed can be used to specifically induce a localized acute oxidative stress in the mitochondria by localizing the fluorophore to the mitochondrial matrix and exposing animals to the excitation spectrum of KillerRed (green light) (*22*). We also induced mitochondrial stress by overexpressing the mitochondrial-binding polyglutamine (polyQ) tract Q40 (*10*). The introduction of mitochondrial stress in CEPsh glia using both methods resulted in a similar activation of UPR^MT^ in peripheral tissue as *jmjd-1.2* overexpression (fig. S1, B to D). Furthermore, we verified our results using an alternative CEPsh glia–specific promoter recently identified using a single-cell RNA sequencing (RNA-seq) dataset (fig. S1E) (*23*). Last, the distal activation by CEPsh glia was dependent on intact glial cells, as ablating the cells using the mutant *vab-3* (*24*) abrogated the effect (fig. S1F). Together, these data indicate that CEPsh glia not only can communicate mitochondrial stress in a nonautonomous manner but also can directly sense mitochondrial stress. These studies also highlight the utility of the *jmjd-1.2* overexpression line as a viable mimetic for nonautonomous communication downstream of mitochondrial stress in glial cells without the caveats of the potentially detrimental effects of mitochondrial dysfunction in neural cells (*10*).

**Fig. 2.**
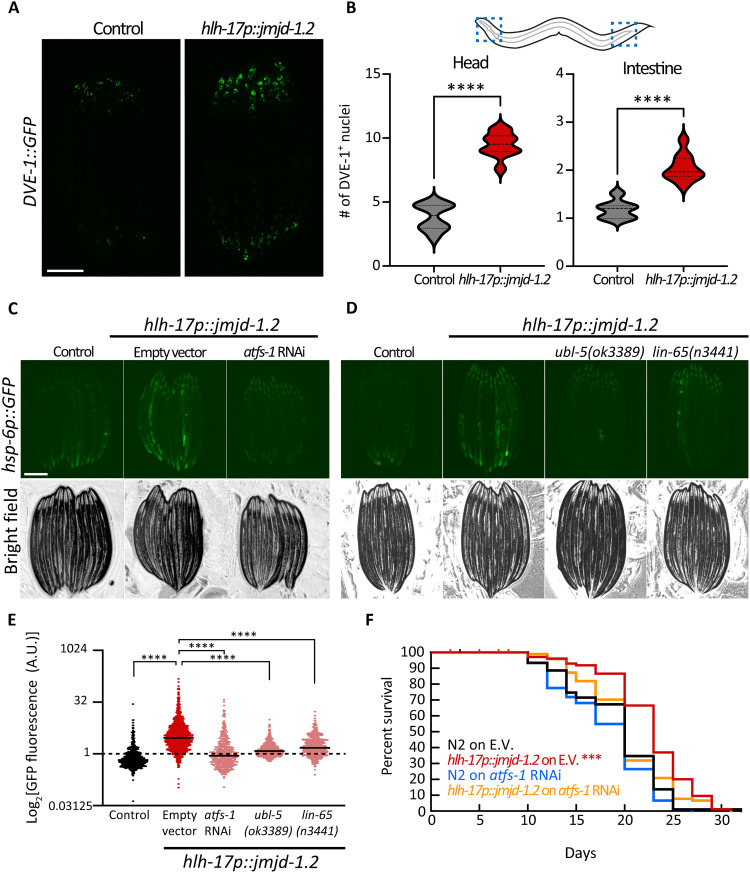
Nonautonomous activation of UPR^MT^ in the periphery depends on cell-autonomous regulators of the pathway UPR^MT^. (**A**) Representative fluorescent micrograph of UPR^MT^ translational reporter worms (DVE-1::GFP) expressing *jmjd-1.2* under the CEPsh glia (*hlh-17*) promoter at day 1 of adulthood. Scale bar, 250 μm. (**B**) The number of DVE-1::GFP foci (i.e., nuclei) was counted in a defined area per worm (*n* > 30), which we called “head” and distal “intestine” as shown in schematic. Significant changes were assessed relative to control by unpaired Student’s *t* test, ****P < 0.0001. (**C** and **D**) Representative fluorescent micrograph of UPR^MT^ reporter worms (*hsp-6p::GFP*) expressing *jmjd-1.2* either knocked down (C) or knocked out (D) for key regulators in UPR^MT^ activation at day 1 of adulthood and quantified in (**E**), as in Fig. 1E (*n* > 450). One-way ANOVA with Tukey’s multiple comparisons test. n.s., not significant; ****P < 0.0001. See also fig. S2. (**F**) Survival of animals expressing *jmjd-1.2* in CEPsh glia grown on either control (E.V.) or *atfs-1* RNAi compared to N2 on E.V. (control); ***P < 0.001. Other conditions are not significantly different from control.

We sought to further decipher the underlying mechanisms of the cell nonautonomous activation triggered by CEPsh glia. First, we tested whether the UPR^MT^ transcriptional program mediated by DVE-1/UBL-5 is also induced upon glial UPR^MT^ induction (*25*, *26*). DVE-1 is a transcription factor, acting with UBL-5 and mediating UPR^MT^ to alleviate stress in the mitochondria (*25*). We found that inducing the UPR^MT^ in CEPsh glia caused an increase in the DVE-1::GFP signal both in the head and the intestinal region of the worm (Fig. 2, A and B). Using the fluorescent reporter *hsp-6p::GFP*, we also found that the activation of the distal UPR^MT^ in response to glial signals depends on three different transcriptional programs of the UPR^MT^: ATFS-1, UBL-5/DVE-1, and LIN-65/MET-2 (Fig. 2, C to E) (*27*). Intact UPR^MT^ was also required for the longevity phenotype, as knocking down the transcription factor ATFS-1, which suppressed UPR^MT^ activation, also abrogated the life-span extension of the glial *jmjd-1.2* animals (Fig. 2F). Collectively, these results indicate that the canonical components of the UPR^MT^ pathway are required for the peripheral response of cell nonautonomous communication from CEPsh glia.

### Activation of UPR^MT^ in glia rewires multiple cellular processes

**Fig. 3.**
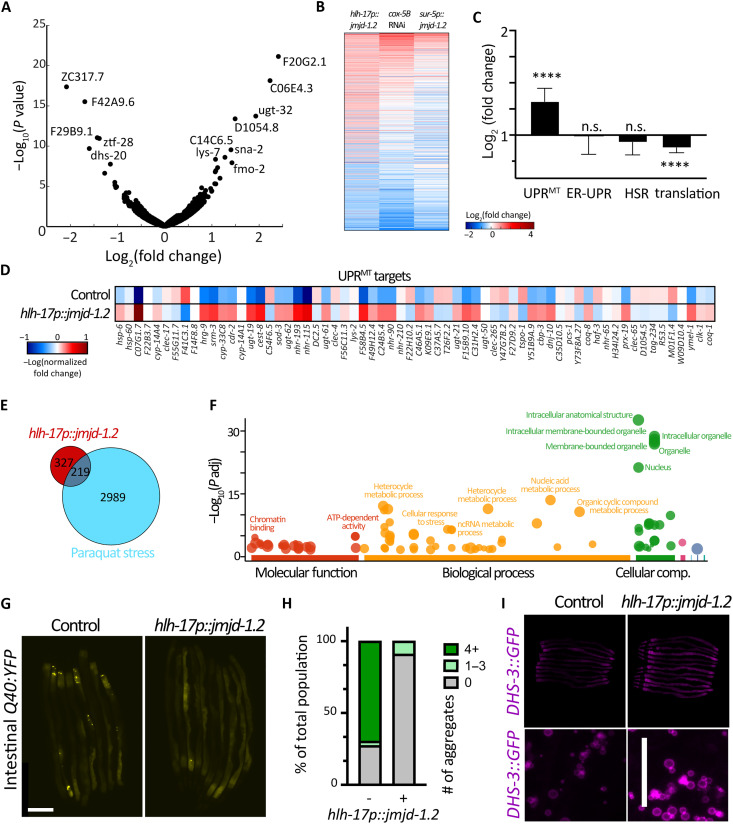
Activation of *jmjd-1.2* in CEPsh glia reduces protein aggregation, increases lipid content in the intestine, and triggers the UPR^MT^ transcriptional program. (**A**) Changes in gene expression in CEPsh glia expressing *jmjd-1.2* as compared to N2 control animals are represented in a volcano plot (for RNA-seq analysis, data are compared using two biological replicates for N2 control and three biological replicates for *hlh-17p::jmjd* = 1.2). (**B**) Differentially expressed genes in glial *jmjd-1.2* worms compared to their change in published datasets of mitochondrial stress induced by electron transport chain knockdown by RNAi (*cox-5B* RNAi) or overexpression of *jmjd-1.2* in all worm tissues (*17*). (**C**) The average change in gene expression of the indicated gene groups (see Methods) as compared to N2 control animals. UPR^MT^ genes are shown in (**D**). HSR, heatshock response. (**E**) Overlap of induced genes in glial *jmjd-1.2* worms with genes induced in paraquat stress. (**F**) Gene enrichment analysis was plotted using gProfiler (*61*). (**G**) Representative fluorescent micrographs of protein aggregation (Q44::YFP) in the intestine of worms expressing *jmjd-1.2* under the CEPsh glia (*hlh-17)* promoter at day 3 of adulthood. Scale bar, 250 μm. (**H**) Quantification of number of fluorescent foci (i.e., aggregates) per worm using Fiji local extrema analysis (*63*) (*n* < 30). (**I**) Representative fluorescent images of lipid droplets reporter animals (*DHS-3::GFP*), either in control animals or animals expressing *jmjd-1.2* in CEPsh glia cells at day 1 of adulthood. Images of aligned whole worms (*n* > 10) were acquired on a stereomicroscope (top) to visualize whole-animal changes in lipid droplet levels, and via high-resolution compound microscopy (bottom) to visualize size and morphology of lipid droplets. Quantification in fig. S3E.

To examine more global changes that occur upon activation of the UPR^MT^ in CEPsh glia, we profiled gene expression changes using whole-animal bulk RNA-seq. We observed many changes in gene expression in glial *jmjd-1.2* animals with 547 significantly up-regulated genes and 413 down-regulated genes compared to a wild-type control (Fig. 3A). As expected, gene expression changes show large similarities to other UPR^MT^ paradigms including electron transport chain inhibition via *cox-5B* RNA interference (RNAi) (*28*) or whole-animal overexpression of *jmjd-1.2* (Fig. 3B) (*17*). Animals with glial *jmjd-1.2* overexpression showed an overall increase in UPR^MT^ genes (Fig. 3, C and D), while other stress pathways did not show a difference. Moreover, we observed a decrease in the expression of genes involved in translation and ribosome biology (Fig. 3C), a characteristic of stress responses meant to alleviate the protein burden on the organelle (*29*). We also observed an overrepresentation of genes involved in the response to oxidative stress, with ~40% of the transcriptional response overlapping with the response to paraquat (Fig. 3E), consistent with our data demonstrating that animals with *jmjd-1.2* induction in CEPsh glia exhibit increased resilience to oxidative stress.

Further enrichment analysis of the up-regulated genes for different gene groups revealed an overrepresentation of genes involved in organellar processes, chromatin-related pathways, metabolic processes, and stress pathways (Fig. 3F). To validate a physiological outcome of the increase in genes involved in stress response and protein homeostasis, we measured the capacity of animals to clear aggregation-prone proteins. Specifically, we measured the aggregation of a polyQ tract in intestinal cells (*30*), which showed a reduction in aggregation upon UPR^MT^ activation in glia both at mid-age (day 5; Fig. 3, G and H) and in early adulthood when exposed to hypertonic stress (fig. S2A) (*31*). In addition, our gene expression analysis suggested that there is also a rewiring of metabolic pathways, including lipid-related genes, which we validated directly by observing an increase in lipid droplet levels and lipid content in the intestine using both the neutral lipid dye BODIPY (fig. S2, B and C) and the lipid droplet marker DHS-3::GFP (Fig. 2I and fig. S2, D and E) (*32*). These data, collectively with intestinal imaging of *hps-6p::GFP*, polyQ, and lipid content, suggest that glial *jmjd-1.2* triggers a transcriptional program across the animal, which affects UPR^MT^, protein homeostasis, and lipid levels.

### Glia use SCVs to communicate with other tissues

The coordination of a whole animal response upon UPR^MT^ activation in CEPsh glia likely requires the transmission of a specific signal(s). The transfer of information between neurons and from neurons relies on distinct secretion pathways, with different encapsulated cargo. Previously, we found that activating the UPR^MT^ in neurons can trigger the UPR^MT^ in the intestine, using the secretion of DCVs (*8*, *10*, *11*), a pathway used for the secretion of polypeptide hormones and neuropeptides. These are synthesized as precursors and packaged into DCVs, processed, and depend on the Ca2^+^-dependent activator UNC-31/CAPS (calcium-dependent activator protein for secretion) protein for secretion (*33*–*35*). In contrast, neurotransmitters, which are generally packaged in SCVs, were not found to be involved in the activation of UPR^MT^ by neurons (*10*), and their release is dependent UNC-13/Munc13 (*36*).

**Fig. 4.**
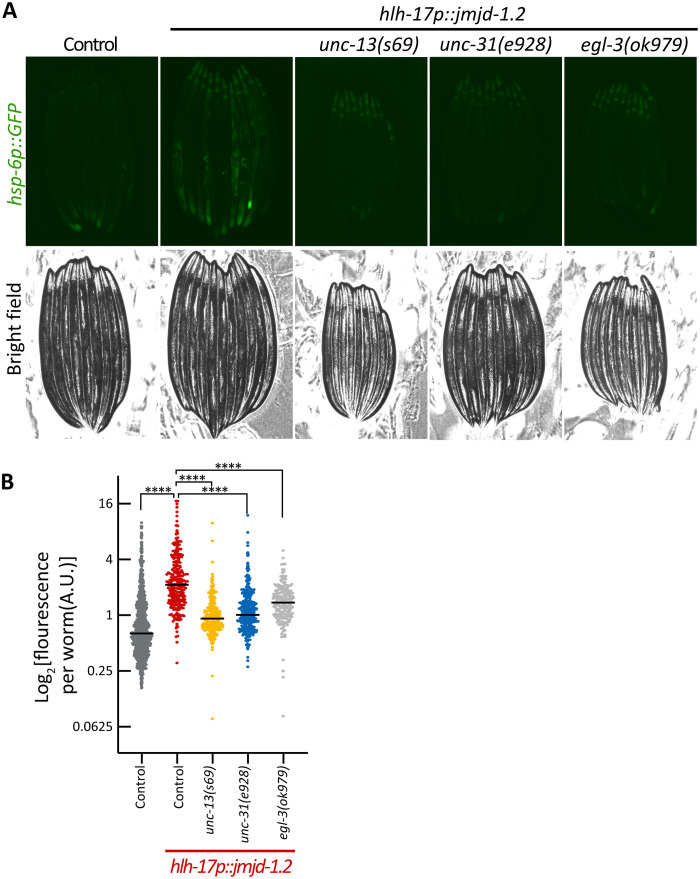
Cell nonautonomous activation of the UPR^MT^ in the intestine depends on the secretion of SCVs, DCVs, and neuropeptide processing. (**A**) Representative fluorescent micrographs of UPR^MT^ reporter worms (*hsp-6::GFP*) for animals with mutations in the secretion of SCVs (*unc-13*), DCVs (*unc-31*), and neuropeptide processing (*egl-3*) at day 1 of adulthood. Scale bar, *25*0 μm. (**B**) Quantification of UPR^MT^ reporter worms (*hsp-6::GFP*) as in Fig. 1E (*n* > 200). ****P < 0.0001.

We examined whether the cell nonautonomous activation from CEPsh glia depends on the exocytosis of DCVs and/or SCVs, by mutating components important for their exocytosis, *unc-31* and *unc-13*, respectively. We found that the activation of UPR^MT^ in intestinal cells depends on both functional UNC-13 and UNC-31 and on neuropeptide processing via EGL-3 as visualized by the loss of intestinal *hsp-6p::GFP* induction in these mutants (Fig. 4, A and B). We also tested for another known signaling molecule required for nonautonomous communication of mitochondrial stress from neurons, the WNT signal EGL-20/WNT5A (*11*). Similar to neuronal signaling, we find that *egl-20* is also required for the distal activation of *hsp-6p::GFP* initiated from glia (fig. S3). Together, our results indicate that glial *jmjd-1.2* activation shares similar genetic requirements in secretion pathways with that of neuronal UPR^MT^ activation with the additional requirement of SCVs (*unc-13*). The requirement of SCVs is perhaps the most unexpected because it clearly differentiates glial signaling from neuronal signaling, as SCVs were previously shown to be dispensable for the nonautonomous communication from neurons. We therefore hypothesize that glia communicate to neurons via *unc-13* (SCVs) and neurons communicate to the periphery via *unc-31* (DCV) signaling.

**Fig. 5.**
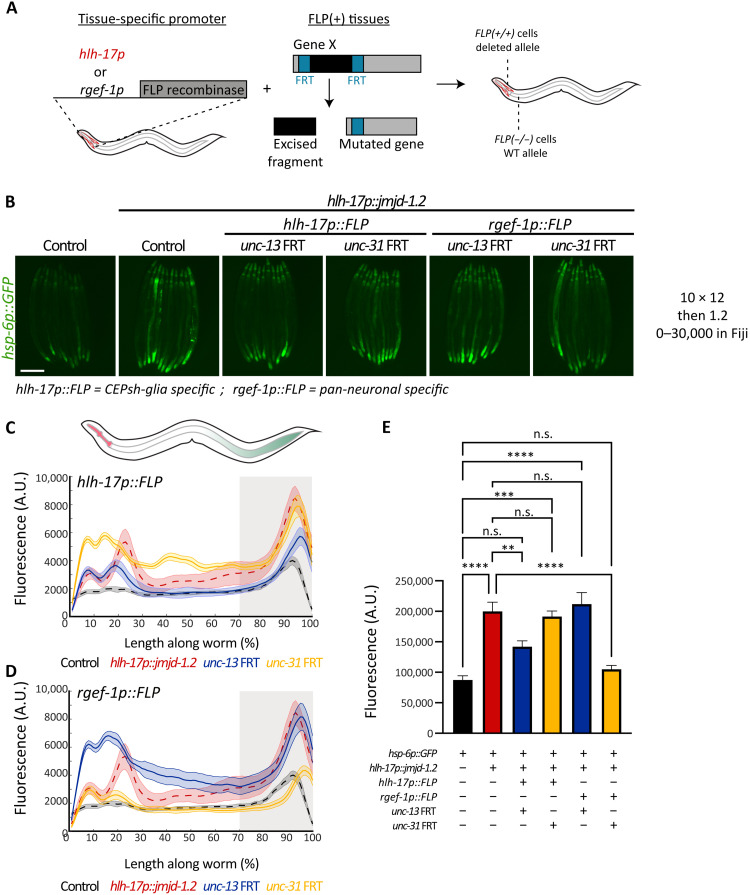
CEPsh glia require functional UNC-13 (SCV), while neurons require UNC-31 (DCV), to activate UPR^MT^ in the periphery. (**A**) Schematic of spatial mutation strategy. Expression of the flipase FLP D5 under the CEPsh glial-specific (*hlh-17p*) or pan-neuronal (*rgef-1p*) promoters, in combination with an independent FRT (FLP recognition target) allele of a gene of interest results in a tissue-specific mutation. (**B**) Representative fluorescent micrographs of the indicated strains at day 1 adult animals. Scale bar, *25*0 μm. (**C** and **D**) Median spatial profiles of the indicated animals (see Methods), for depletion in CEPsh glial cells (C) or neuronal cells (D) quantified with large particle biosorter (*n* > 50). The integrated fluorescence intensity of the 30% most posterior portion of the animals was calculated and plotted in (**E**). One-way ANOVA with Tukey’s multiple comparisons test, ***P < 0.001 and ****P < 0.0001.

To directly test this hypothesis and uncover in which tissues (glia versus neurons) *unc-13* and *unc-31* participate in the UPR^MT^ signaling, we knocked out *unc-13* and *unc-31* in a tissue-specific manner using the flippase/flippase recognition target (FLP/FRT) system (*37*). This system allows deleting a portion of a gene by expressing the flipase, FLP D5, in specific tissues of interest (CEPsh glia versus pan-neuronal) in combination with an allele of the gene of interest (*unc-13* or *unc-31*) to which two copies of the FRT sequence have been introduced using CRISPR-Cas9 genome editing (Fig. 5A and fig. S4, A to C). The resulting animals have spatially distinct genotypes, with a defect in the secretion of SCVs or DCVs exclusively in one tissue. Notably, we observed that knocking out *unc-13* specifically in glia, or *unc-31* specifically in neurons, was able to alleviate the activation of the UPR^MT^ in the intestine (Fig. 5, B to E). Moreover, rescuing *unc-13* specifically in neurons, under the *snb-1* promoter, was not able to restore the activation of UPR^MT^ in the periphery (fig. S4D). Last, we found that knocking out *unc-13* specifically in glia suppressed the life-span extension of glial *xbp-1s* animals, whereas knockout of *unc-31* in glia had no effect (fig. S5). These results, together with our previous work on neuronal activation of the UPR^MT^, indicate a linear model, in which glia secrete a molecule by SCVs (*unc-13*), that is perceived by neurons, which then relay the information to the periphery via DCVs (*unc-31*). Glial communication to neurons through UNC-13 is necessary for the life-span extension found in these animals.

### Activation of UPR^MT^ in glia improves neuronal protein homeostasis

**Fig. 6.**
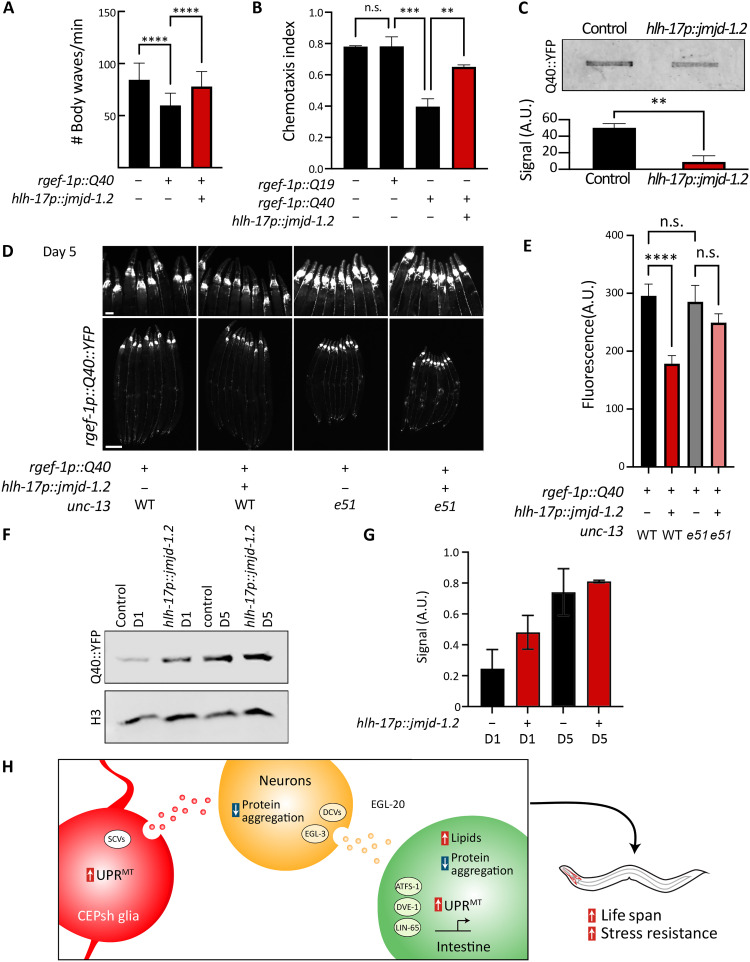
Glial *jmjd-1.2* rescues protein aggregation in neurons in an HD model via SCVs. (**A**) Thrashing of animals expressing the aggregating polyQ tract Q40, with and without glial *jmjd-1.2*, measured using WormTracker (*64*) (*n* > 100). (**B**) Chemotaxis index of worms toward benzaldehyde (*n* > 200). (**C**) Filter retardation assay for Q40::YFP (*39*), with and without *glial jmjd-1.2* (top) and its quantification using integrated intensity measurements in Fiji (bottom). Data are representative of three independent biological replicates. (**D**) Representative fluorescent micrographs of Q40::YFP for the annotated genotypes on day 5 of adulthood. See fig. S6 for images of worms at day 1. WT, animals expressing wild-type copy of *unc-13*; e51, animals expressing loss of function mutant *unc-13(e51)*. (**E**) Integrated fluorescence intensity measurements of Q40::YFP in the head region of the animals (*n* > 30) using Fiji. One-way ANOVA with Tukey’s multiple comparisons test; **P < 0.01, ***P < 0.001, and ****P < 0.0001. (**F**) Representative blot of neuronal Q40::YFP protein at days 1 and 5 of adulthood in control (N2) and glial *jmjd-1.2* (*hlh-17p::jmjd-1.2*) animals. Total Q40::YFP expression was measured via standard Western blots in whole worm lysates using a standard anti-GFP antibody. Signal intensity was quantified using integrated intensity measurements in Fiji in (**G**) and normalized to an H3 load control. Measurements in (G) were performed on two biological replicates, and data are presented as means ± SD. (**H**) Model of communication from glial cells to peripheral tissues. CEPsh glia use SCVs upon UPR^MT^ activation to signal to neurons, which reduce protein aggregation and use DCVs, neuropeptide processing, and a WNT ligand to drive protein homeostasis and metabolic changes in the periphery.

Considering our model whereby activation of glial UPR^MT^ results in a glia-to-neuron signal, we next questioned whether glial UPR^MT^ is able to drive improved protein homeostasis in neurons. Our data and previous studies (*10*) showed that nonautonomous UPR^MT^ signals can promote protein homeostasis in the target tissues. Moreover, the importance of protein homeostasis in neurons is best illustrated by neurodegenerative diseases, in which protein aggregation occurs (*5*), of which glial health has been suggested as a critical factor in maintenance of neuronal fitness (*14*). Thus, we used the well-established HD model in *C. elegans*, in which a 40-repeat polyglutamine tract (Q40) is expressed in all neuronal cells. Q40 is able to bind directly to mitochondria and has been shown to also affect mitochondria directly in the same tissue (*10*, *38*). We activated the UPR^MT^ in glia and measured a battery of phenotypes associated with the neuronal HD model. Notably, activating UPR^MT^ in glia was able to rescue the thrashing and chemotaxis defects observed in neuronal Q40 animals (Fig. 6, A and B, and fig. S6).

The polyQ model is also useful for directly measuring protein aggregation, as the Q40 tract is tagged with a YFP. We imaged worms and observed decreased aggregation of the protein in late age (Fig. 6, D and E, and fig. S6C). We further verified the aggregation using a filter-trap assay (*39*), where we observed less aggregation when activating the UPR^MT^ in glia (Fig. 6C). This decline in aggregation is likely due to an increase in proteostasis machinery as suggested in our transcriptomics data (Fig. 3) rather than a general decrease in protein expression, as we found that glial *jmjd-1.2* overexpression did not result decreased neuronal Q40 expression (Fig. 6, F and G). Next, we tested whether these effects are dependent on SCVs. We find that intact UNC-13 signaling was required for alleviating the aggregation in neurons, such that mutants of *unc-13* show failure of decreasing Q40 aggregation in neurons when overexpressing *jmjd-1.2* in CEPsh glia (Fig. 6, D and E). Last, we asked whether this protection by glia is a general attribute of CEPsh glia, by activating other protein homeostasis pathways in CEPsh glia and testing their ability to improve protein homeostasis in neurons. To this end, we used our previous model of activating the UPR^ER^ in CEPsh glia, using an overexpression of the spliced version of the transcription factor *xbp-1 s* (*16*). We were not able to observe any beneficial effects (fig. S6D), highlighting that the ability of glia to improve neuronal protein homeostasis is unique to the UPR^MT^.

## DISCUSSION

In this study, we discovered a role for a small subset of glial cells in sensing mitochondrial stress and signaling a beneficial cell-nonautonomous communication to neurons. This signal drives a coordinated change in mitochondrial protein homeostasis across tissues, via a relay-cellular pathway mediated by neurons. We found that communication from glia occurs as a two-step process: The astrocyte-like CEPsh glial cells secrete a signal via SCVs, and, subsequently, neurons relay the signal to peripheral tissues by using DCVs and the WNT ligand EGL-20 (Fig. 6F).

We found that different subpopulations of glial cells can regulate longevity and coordinate the communication of mitochondrial proteotoxic stress across the tissues of the animal. We were most struck by the CEPsh glia, a cell type that includes only four individual cells that resulted in multiple physiological benefits, including increased life span and stress resistance, with full dependency on the functionality of the regulators involved in the UPR^MT^. Our efforts to uncover and disentangle the requirement of genes involved in intercellular signaling revealed that CEPsh glia use SCVs to signal the mitochondrial proteotoxic stress. Mammalian astrocytes were suggested to possibly express Munc13, in cultured and freshly isolated astrocytes (*40*, *41*), as similarly observed in RNA-seq data from isolated CEPsh glia in nematodes (fig. S4E) (*42*), and our findings may provide the context in which these vesicles are used.

Our dissection of the functional importance of different genes in communicating the UPR^MT^ from glia to the intestine suggests that the signal is mediated by neurons. We were thus inspired to test whether the internal state of neurons themselves is altered upon receiving the UPR^MT^ from glia. We found that glial UPR^MT^ can promote protein homeostasis in neurons and protect them from the detrimental effects of polyQ expression. Conventionally, neurons have been at the center of studying neurodegenerative diseases, and glial cells, such as astrocytes, have been implicated in neurogenerative diseases, mostly by ablation experiments showing that glial cells can exacerbate disease pathology (*13*). Thus, whether the loss of glial cells directly caused disease phenotypes was not understood, and most studies concluded that the breakdown of neuronal function was the true culprit. However, our study supports a redefining role of astrocytes from simply supporting neuronal cells to playing an active role in sensing stress and communicating a beneficial signal to neurons, which can then mediate a whole-organism response to stress. These findings have potentially huge ramifications for the study of neurodegenerative diseases, as it shifts attention to glial cells and asks the question of whether glial cells should be the true target for potential therapies in neurodegenerative disorders. Increased activity of astrocytes identified by those with higher levels of the glutamate transporter, GLT-1, was found to preserve cognitive function in patients with AD (*43*).

An important question raised by our study is whether CEPsh glia can uniquely sense mitochondrial stress. Our data show that activation of the UPR^MT^, but not the UPR^ER^, in CEPsh glia resulted in increased neuronal protein homeostasis. There are several plausible explanations for this: First, it is possible that a distinct glial population is required for communicating ER stress to neurons, whereas CEPsh glia can only communicate ER stress directly to the periphery. Other studies have shown that neuronal cells differ in their capacity to sense and signal distinct types of UPR^ER^ signals (*44*), and it is entirely possible that additional subsets of glia can signal ER stress and that only a specific subtype can signal directly to neurons. Another possibility is that mitochondrial stress signals are uniquely able to activate protein homeostasis machinery adept at clearing polyQ aggregates, whereas other stress signals may activate protein homeostasis machinery for other forms of protein dysfunction. Previous studies have shown that polyQ aggregates can bind mitochondria and induce the UPR^MT^ (*10*), suggesting that there are organelle-specific effects of certain classes of aggregates. Last, it is entirely possible that glial UPR^ER^ signals do not involve communication with neurons, neither to promote neuronal health nor to use neurons as the intermediate signal to the periphery. Glial UPR^ER^ signals were independent of neuronal signaling, and *unc-31* rescue solely in glial cells were able to drive nonautonomous activation of UPR^ER^ in glial *xbp-1 s* animals (*16*). In comparison of our data with the previously published study, it seems likely that, for UPR^ER^ signals, glial cells can communicate directly to the periphery via DCVs, while, for UPR^MT^ signals, glia first communicate to neurons through SCVs and then neurons communicate to the periphery through DCVs. Thus, by bypassing neurons, it is possible that glial UPR^ER^ fails to improve neuronal proteostasis. Perhaps the most important lesson from these studies is that the same glial cells, CEPsh glia in this case, can use completely divergent signaling mechanisms based on which type of stress is sensed.

Our data whereby SCV release (*unc-13*) but not DCVs (*unc-31*) by glial cells is required for the glial UPR^MT^ signal add further evidence that signaling of stress is context dependent. First, it suggests that, even if the *hlh-17p* may potentially express in very low levels in other neural cells, the glial contributions for nonautonomous stress signaling are more significant as glial-specific *unc-13* knockout completely suppressed life-span extension found in glial *jmjd-1.2* animals. In addition, glial signaling of UPR^ER^, in contrast to UPR^MT^, required DCV release (*unc-31*), but not SCVs (*unc-13*) (*16*). Moreover, glial UPR^ER^ signaling was seemingly independent of neurons, whereby our data suggest that UPR^MT^ signals follow a glia to neuron to periphery signature. It is also entirely possible that neurons also up-regulate UPR^MT^ during this glia to neuron to periphery transmission. While we could not directly measure UPR^MT^ activation in neurons through high-resolution microscopy, our biosorter data suggest that there is a high level of green fluorescent protein (GFP) signal in the head that is suppressed upon *unc-13* knockout, which could potentially be neuronal UPR^MT^ activation.

Together, these studies suggest that similar to neurons, glia have the capacity to sense and signal various different types of stress and use divergent pathways to communicate with the periphery based on the type of stress. While seemingly far-fetched, a recent study has shown that neurons can elicit entirely different downstream responses in the periphery solely based on which neurotransmitter is used. Specifically, dopaminergic and serotonergic circuits, both experiencing identical stress in the form of UPR^ER^ activation, have the potential to induce lipid remodeling or protein homeostatic responses in the periphery, despite the origin of the signal, *xbp-1 s*, being identical (*44*). Thus, it is entirely possible that glia can also initiate different signaling paradigms using unique “gliotransmitters” based on the type of stress that is being sensed. Moreover, it is also possible that certain glial subtypes benefit from UPR^MT^ activation, while others do not: Specifically, we found that pan-glial *jmjd-1.2* overexpression resulted in a much milder life-span extension compared to *jmjd-1.2* overexpression in CEPsh glia alone. Overexpression of *jmjd-1.2* may have negative consequences in some glial cells, which negate any positive benefits from other glial subtypes. In UPR^ER^ activation, *xbp-1 s* overexpression in muscle cells resulted in decreased life span, suggesting that hyperactivation of stress responses can be detrimental in some instances (*6*). An alternative hypothesis is that glial UPR^MT^ signatures can only be transmitted via CEPsh glia, and, while the *ptr-10* promoter includes these neurons, expression in CEPsh glia via the *prt-10* promoter is much more limited in comparison to the *hlh-17* promoter. Overall, these differences do highlight that, on an organismal level, the capacity to compartmentalize whole organism-stress responses by using divergent signaling methods would be useful to avoid up-regulating unnecessary, or even detrimental, pathways irrelevant to the stress that is now being experienced.

Last, we found that glia have the ability to improve protein homeostasis not only on the physiological level (thrashing, chemotaxis) but also on the molecular level, measuring the aggregating protein itself. Similarly, recent work in mice found that astrocytes, through activation of the Janus kinase 2–signal transducer and activator of transcription 3 pathway, are able to clear protein aggregates in neurons in a mutant huntingtin mouse model (*45*), highlighting our findings of a UNC-13–related improvement of protein homeostasis in the glial-neuron axis. Of note, UNC-13/Munc13 has previously been linked also to another neurodegenerative disease, amyotrophic lateral sclerosis (ALS). Genome-wide association studies linked UNC-13A to ALS as mutations associated with higher susceptibility and shorter survival (*46*–*48*) in individuals with ALS, a link that is evolutionarily conserved in *C. elegans* (*49*). Whether the involvement of UNC-13 in ALS stems from its glial functions remains to be explored.

In summary, our work not only highlights the importance of glial cells in communicating mitochondrial proteotoxic stress and coordinating between tissues but also mechanistically mapped SCVs as the secretion pathway used from these cells. Our study places CEPsh glia as upstream regulators of coordination of stress responses and underlines their role as a major signaling hub that can affect cellular and organismal homeostasis. In combination with our findings on protein aggregation in neurons, our results underscore the importance on examining the roles and mechanisms used by glial cells to regulate protein homeostasis within the nervous system and in the periphery. Advancing our knowledge of how glial cells regulate protein homeostasis in the context of mitochondrial stress will be critical in ongoing efforts to understand the glia-neuron communication axis in neurodegenerative diseases and in aging.

## METHODS

### Strains and maintenance

All *C. elegans* strains are derivatives of the Bristol N2 wild-type strain from the Caenorhabditis Genetics Center (CGC) and are listed in Table 1. All worms were grown at 15° to 20°C on NGM (nematode growth medium) agar plates, fed with OP50 *Escherichia coli* B strain as a food source, for general maintenance, and handled as previously described (*50*). For all experiments, worms were switched to HT115 *E. coli* K12 strain after synchronization using bleaching. Worms were grown for at least three generations at 20°C on OP50 before synchronization to acclimate to the temperature. HT115 bacteria were carrying the pL4440 empty vector control or expressing double-stranded RNA containing the sequence against a target gene, as specified in each corresponding figure legend. RNAi clones against *atfs-1* and *daf-2* were acquired from the Vidal RNAi library (*51*) and verified using Sanger sequencing. All experiments were performed on age-matched animals synchronized using a standard bleaching protocol, to day 1 of adulthood, or aged to later ages, as specified. Synchronization was achieved by washing animals fed with OP50 *E. coli* with M9 solution (22 mM KH_2_PO_4_ monobasic, 42.3 mM Na_2_HPO_4_, 85.6 mM NaCl, and 1 mM MgSO_4_), bleached using a solution of 1.8% sodium hypochlorite and 0.375 M KOH diluted in DDW, for 4 to 5 min, until all carcasses were digested. Intact eggs were then washed four times with M9 solution, and intact eggs were verified under the microscope after seeding.

**Table 1.** Strains and plasmids used in this study. UTR, untranslated region.

Identifier	Genotype	Source
N2	N2, wild type	CGC
JSD1038	snb-1p::UNC-13 L, unc-13(s69)	Dittman lab
AGD1395	uthIs393[vha-6p::Q40::YDP + rol-6(su1006)]	Integrated from (*65*)
AGD1900	egl-3(ok979)V	VC671 (CGC)
AGD1924	vab-3(e648)X	CB648 (CGC)
AGD1988	zcIs13[hsp-6p::GFP]V	SJ4100 (CGC)
AGD1989	unc-13(s69) I	EG9631 (CGC)
AGD2016	lin-65(n3441)	MT13232 (CGC)
AGD2038	uthIs506(hlh-17p::jmjd-1.2::tbb-2 3′UTR, myo-2p::tdTomato), zcIs13(hsp-6p::GFP); egl-3(ok979)V	This study
AGD2077	rmIs110 [rgef-1p::Q40::YFP]	AM101 (CGC)
AGD2225	unc-31(e928)IV	CB928 (Jin lab)
AGD2739	uthIs506(hlh-17p::jmjd-1.2::tbb-2 3′UTR, myo-2p::tdTomato)	This study
AGD2771	uthIs506(hlh-17p::jmjd-1.2::tbb-2 3′UTR, myo-2p::tdTomato), zcIs13[hsp-6p::GFP]V	This study
AGD2772	uthIs506(hlh-17p::jmjd-1.2::tbb-2 3′UTR, myo-2p::tdTomato), zcIs39[dve-1p::dve-1::GFP] II	This study
AGD2800	uthEx951(hlh-17p::Q40::tbb-2 3′UTR, myo-2p::tdTomato), line #2) zcIs13[hsp-6p::GFP]	This study
AGD2871	uthIs506[hlh-17p::jmjd-1.2::tbb-2 3′UTR, myo-2p::tdTomato]; zcIs13[hsp-6p::GFP]; egl-20(n585)	This study
AGD2887	uthIs506(hlh-17p::jmjd-1.2::tbb-2 3′UTR, myo-2p::tdTomato), zcIs13[hsp-6p::GFP]V, vab-3(e648)X	This study
AGD2902	uthIs506[hlh-17p::jmjd-1.2::tbb-2 3′UTR, myo-2p::tdTomato]; zcIs13[hsp-6p::GFP]; unc-13(s69) I	This study
AGD2909	egl-20(n585) IV	MT1215 (CGC)
AGD2923	uthIs506[hlh-17p::jmjd-1.2::tbb-2 3′UTR, myo-2p::tdTomato];zcIs13[hsp-6p::GFP]; ubl-5(ok3389)I	This study
AGD2925	uthIs506(hlh-17p::jmjd-1.2::tbb-2 3′UTR, myo-2p::tdTomato); zcIs13[hsp-6p::GFP];unc-31(e928)IV	This study
AGD2970	uthIs506(hlh-17p::jmjd-1.2::tbb-2 3′UTR, myo-2p::tdTomato), ldrIs1[dhs-3p::dhs-3::GFP + unc-76(+)]	This study
AGD3023	uthIs506(hlh-17p::jmjd-1.2::tbb-2 3′UTR, myo-2p::tdTomato), line #1 x dgEx80[pAMS66 vha-6p::Q44::YFP + rol-6(su1006) + pBluescriptII]	This study
AGD3047	rmIs110 [rgef-1p::Q40::YFP], uthIs506(hlh-17p::jmjd-1.2(phf-8)::tbb-2 3′UTR, myo-2p::tdTomato)	This study
AGD3048	uthIs511(fig-1p::jmjd-1.2::tbb-2, myo-2p::tdTomato)	This study
AGD3057	uthIs506(hlh-17p::jmjd-1.2::tbb-2 3′UTR, myo-2p::tdTomato), zcIs39[dve-1p::dve-1::GFP]II;; egl-3(ok979)V	This study
AGD3058	uthIs506(hlh-17p::jmjd-1.2::tbb-2 3′UTR, myo-2p::tdTomato), zcIs39[dve-1p::dve-1::GFP]II; unc-31(e928)IV	This study
AGD3110	uthIs511(fig-1p::jmjd-1.2::tbb-2, myo-2p::tdTomato), zcIs13[hsp-6p::GFP]V	This study
AGD3185	uthIs517(ptr-10p::jmjd-1.2::tbb-2 3′UTR, myo-2p::tdTomato)	This study
AGD3319	rmIs110 [rgef-1p::Q40::YFP]; unc-13(e51) I	This study
AGD3332	bqSi506 [rgef-1p::FLP D5 + unc-119(+)] IV; unc-31(syb3118, syb3119)	This study
AGD3341	rmIs110 [rgef-1p::Q40::YFP]; unc-13(e51) I; uthIs506(hlh-17p::jmjd-1.2(phf-8)::tbb-2 3′UTR, myo-2p::tdTomato),	This study
AGD3367	bqSi506 [rgef-1p::FLP D5 + unc-119(+)] IV; unc-31(syb3118, syb3119); zcIs13[hsp-6p::GFP]	This study
AGD3378	N2; unc-13p::frt::unc-13::frt; sybIs2974[hlh-17p::NLS-FLP D5::tbb-2 3′UTR;Cbr-unc-119(+)] II; unc-119(ed3);	This study
AGD3379	uthIs440[hlh-17p::xbp-1 s, myo-2p::tdTomato]; rmIs110 [rgef-1p::Q40::YFP]	This study
AGD3411	uthIs506(hlh-17p::jmjd-1.2::tbb-2 3′UTR, myo-2p::tdTomato), zcIs39[dve-1p::dve-1::GFP]II; unc-13(s69) I	This study
AGD3416	uthIs506(hlh-17p::jmjd-1.2::tbb-2 3′UTR, myo-2p::tdTomato), line #1;zcIs13(hsp-6p::GFP)V; sybIs2974[hlh-17p::NLS-FLP D5::tbb-2 3′UTR;Cbr-unc-119(+)] II; unc-119(ed3), unc-13 (syb3090, syb2916)I	This study
AGD3418	uthIs517(ptr-10p::jmjd-1.2::tbb-2 3′UTR, myo-2p::tdTomato), zcIs13[hsp-6p::GFP]V	This study
AGD3442	uthIs506(hlh-17p::jmjd-1.2::tbb-2 3′UTR, myo-2p::tdTomato),;zcIs13(hsp-6p::GFP)V; bqSi506 [rgef-1p::FLP D5 + unc-119(+)] IV; unc-119(ed3), unc-31(syb3118, syb3119)I	This study
AGD3451	sybIs2974[hlh-17p::NLS-FLP D5::tbb-2 3′UTR;Cbr-unc-119(+)] II; unc-119(ed3); syb3684; unc-31(syb3118, syb3119)	This study
AGD3451	sybIs2974[hlh-17p::NLS-FLP D5::tbb-2 3′UTR;Cbr-unc-119(+)] II; unc-119(ed3); syb3684; unc-31(syb3118, syb3119)	This study
AGD3459	uthEx992(hlh-17p::mitoKillerRed:tbb-2, coel::RFP); zcIs39[dve-1p::dve-1::GFP]II	This study
AGD3467	sybIs2974[hlh-17p::NLS-FLP D5::tbb-2 3′UTR;Cbr-unc-119(+)] II;unc-31(syb3118, syb3119); zcIs13[hsp-6p::GFP]	This study
AGD3472	bqSi506 [rgef-1p::FLP D5 + unc-119(+)] IV; unc-119(ed3), unc-13 (syb3090, syb2916)I;zcIs13[hsp-6p::GFP]	This study
AGD3477	snb-1p::unc-13; uthIs506[hlh-17p::jmjd-1.2::tbb-2 3′UTR, myo-2p::tdTomato]; zcIs13[hsp-6p::GFP]; unc-13(s69)	This study
AGD3491	uthIs506(hlh-17p::jmjd-1.2::tbb-2 3′UTR, myo-2p::tdTomato), zcIs13[hsp-6p::GFP]; lin-65(n3441)	This study
AGD3506	bqSi506 [rgef-1p::FLP D5 + unc-119(+)] IV; unc-119(ed3), unc-13 (syb3090, syb2916)I;zcIs13[hsp-6p::GFP]; uthIs506(hlh-17p::jmjd-1.2::tbb-2 3′UTR, myo-2p::tdTomato)	This study
AGD3507	sybIs2974[hlh-17p::NLS-FLP D5::tbb-2 3′UTR;Cbr-unc-119(+)] II;unc-31(syb3118, syb3119); zcIs13[hsp-6p::GFP]; uthIs506(hlh-17p::jmjd-1.2::tbb-2 3′UTR, myo-2p::tdTomato)	This study
RHS104	uthIs393[vha-6p::Q40::YFP + rol-6(su1006)]; uthIs50 6(hlh-17p::jmjd-1.2(phf-8)::tbb-2 3′UTR, myo-2p::tdTomato)	This study
BN507	bqSi294 [hsp16.41p::FRT::mCherry::his-58::FRT::GFP::his-58 + unc-119(+)] II; bqSi506 [rgef-1p::FLP D5 + unc-119(+)] IV	CGC
GF80	dgEx80[pAMS66 vha-6p::Q44::YFP + rol-6(su1006) + pBluescriptII]	CGC
LIU1	ldrIs1[dhs-3p::dhs-3::GFP + unc-76(+)]	Liu lab
PHX3090	unc-13 (syb3090 syb2916)	SunyBiotech
PHX3119	unc-31(syb3118, syb3119)	SunyBiotech
PHX3684	sybIs2974[hlh-17p::NLS-FLP D5::tbb-2 3′UTR;Cbr-unc-119(+)] II; unc-119(ed3); syb3684	SunyBiotech
PHX5532	uthEx992(hlh-17p::mitoKillerRed:tbb-2, coel::RFP)	SunyBiotech
SJ4197	zcIs39[dve-1p::dve-1::GFP]II	CGC
VC2654	ubl-5(ok3389) I	CGC
pRBZ101	hlh-17p::jmjd-1.2::tbb-2 3′UTR	This study
pRBZ102	hlh-17p::Q40-HA::tbb-2 3′UTR	This study
pRBZ103	fig-1p::jmjd-1.2::tbb-2 3′UTR	This study
pRBZ104	ptr-10p::jmjd-1.2::tbb-2 3′UTR	This study
pRBZ105	mir-228p::jmjd-1.2::tbb-2 3′UTR	This study
pES09	mfsd-13.2p::NLS::mCherry::tbb-2 3′UTR	This study
pES06	mfsd-13.2p:jmjd::tbb-2 3′UTR	This study
pES04	hlh-17p::tomm20::KillerRed::tbb-2 3′UTR	This study

Strains were generated by cloning the cDNA of interest under the relevant promoter, using Gibson Assembly (*52*). The cDNAs of *jmjd-1.2* and the polyQ Q40 were amplified from pCM407 (*17*) and pJKD101 (*10*), respectively. The promoters *ptr-10p*, *fig-1p*, and *hlh-17p* were subcloned from pAF12, pAF5, and pAF6 (*16*), respectively. Synthetic DNA of *tbb-2* 3′ untranslated region and *mfsd-13.2* promoter was synthesized by Twist Bioscience. Wild-type N2 strain worms were injected with the construct of interest, along with a *myo-2p::tdtomato or unc-122p::RFP* coinjection marker. Worms were selected under a fluorescent microscope and then integrated by ultraviolet irradiation. Integrated lines were backcrossed eight times to a wild-type N2 strain. For same-orientation FRT insertions, *unc-13* insertions between exons 18 and 19 and between exons 20 and 21 as well as *unc-31* insertions between exons 1 and 2 and between exons 2 and 3 were made using CRISPR-Cas9 by SUNY biotech, and successful cutting was verified using polymerase chain reaction and gel electrophoresis, followed by sequencing (see fig. S5A).

### Microscopy and of UPR^MT^ reporters

Imaging of *hsp-6p::GFP* and DVE-1::GFP fluorescent reporters was done as previously described (*53*). Briefly, animals were grown on standard RNAi plates from hatch at 20°C until day 1 of adulthood. Animals were picked under a standard dissection microscope with white light at random to avoid biased sampling. Then, animals were anesthetized using a 10- to 15-μl drop of 100 mM sodium azide (NaN_3_) on NGM plates with no bacteria and aligned to have the same orientation. Images were captured on a Leica M250FA stereoscope equipped with a Hamamatsu ORCA-ER camera driven by LAS-X software or using an Echo Revolve R4 microscope equipped with an Olympus 4× Plan Fluorite numerical aperture of 0.13 objective lens, a standard Olympus FITC filter (excitation, 470/40; emission, 525/50; dichroic mirror, 560). Bright-field and fluorescent images were acquired, with exposure time and laser intensity matched within experiments. Each micrograph contained 10 individual worms and was independently replicated at least three times.

### Large-particle flow cytometry

To quantify fluorescent reporters, flow cytometry using a Union Biometrica bioSorter (cat. no. 250-5000-000) was done as previously described (*53*). Briefly, staged worms were washed off plates using M9, allowed to settle by gravity, and washed once with M9 to separate from eggs. The signal was collected for time of flight (TOF; length) and extinction (thickness) of animals, along with the GFP and red fluorescent protein (RFP). Data were collected gating for size (TOF and extinction) to exclude eggs. Data are represented as an integrated intensity of fluorescence normalized to the size of the animal using the integrated GFP output and dividing by the extinction and TOF. All data that exceed the measurement capacity of the photomultiplier tube (PMT), calculated as a signal of 65,355, are considered saturated and are censored from the calculation. For spatial profiles, the complete profiles were extracted, and worms were aligned according to their *myo-2p::tdtomato* (red head) signal using MATLAB (MathWorks) and binned into 100 bins to account for differences in animal length (*n* > 50). Then, the average profile and SEM were calculated on binned profiles. For *hsp-6p::GFP* worms, which do not harbor a *myo-2p::tdtomato* coinjection marker, the profiles were aligned according to the GFP signal, with the highest peak of signal defined as the posterior intestine.

### Life-span analysis

Life-span analyses were performed as previously described (*53*). All animals were grown at 20°C on HT115 *E. coli*, with 80 to 120 animals used per condition and scored every day or every other day, starting from day 1 of adulthood. Animals were moved away from progeny onto fresh plates for the first 5 to 7 days until progeny was no longer visible. Animals with bagging, vulval explosions, or other age-unrelated deaths were censored and removed from quantification. At least two independent replicates per condition. Analyses were done using Prism 8 (GraphPad). *P* values were calculated using the log-rank (Mantel-Cox) method.

### Paraquat resistance assay

Resistance to oxidative stress generated by exposure to 100 mM paraquat (Sigma-Aldrich, 36541) (*19*) was done as previously described (*53*), with three biological replicates per condition. Briefly, fresh paraquat was prepared in M9 solution and aliquoted into a flat-bottom 96-well plate, with five animals per well, with 12 wells per strain (*n* = 60). Every 2 hours, animals were scored for death in each well. For all paraquat assays, *daf-2* RNAi is used as a positive control as *daf-2* animals exhibit significant resistance to oxidative stress using this assay (*53*).

### RNA-seq and analysis

Animals were synchronized using a standard bleaching protocol, and all RNA collection was performed at day 1 of adulthood fed with HT115 bacteria. A total of 1000 to 2000 day-1 animals were harvested using M9, and animals were pelleted by centrifugation. M9 was subsequently aspirated and replaced with TRIzol solution. Worms were freeze-thawed three times with liquid nitrogen, and a ~30-s vortexing was performed before each refreeze. After the final thaw, chloroform was added at a 1:5 ratio (chloroform:TRIzol), and aqueous separation of RNA was performed via centrifugation in a heavy-gel phase-lock tube (VWR, 10847--802). The aqueous phase was collected and mixed with isopropanol at a 1:1 ratio, and, then, RNA purification was performed using the QIAGEN RNeasy Kit as per the manufacturer’s directions. Library preparation was performed using the Kapa Biosystems mRNA Hyper Prep Kit. Sequencing was performed at the Vincent J. Coates Genomic Sequencing Core at the University of California, Berkeley, using an Illumina NovaSeq SP SR100. For N2 control and *hlh-17p::jmjd-1.2*, two or three biological replicates were done, respectively. Reads were aligned using HISAT2 (version 2.2.1) (*54*) with WBcel235 as the reference genome and quantified using featureCounts (*55*), and differentially expressed genes were calculated using DESeq2 (*56*). For gene group analyses, mitochondrial UPRs were defined as previously annotated by Soo *et al.* (*57*); ER-UPR (GO:0030968), heatshock response (HSR) (GO:0009408), and translation (GO:0006412) genes were defined using Gene Ontology (*58*, *59*). Comparison to other gene expression collections was done using WormExp (*60*). Gene enrichment was done using gProfiler (*61*) and GOrilla (*62*) for significantly changing genes (*P* < 0.05). Plots were generated using MATLAB (MathWorks).

### BODIPY staining

Neutral lipid measurements were done using BODIPY 409/503 staining as previously described (*32*), with three biological replicates. Briefly, worms were synchronized by bleaching, grown on empty vector (EV), and harvested at larval stage 4 (L4). Worms were washed three times in M9 buffer. Then, 4% paraformaldehyde was added and incubated for 15 min to fixate the samples. Next, the sample was frozen in liquid nitrogen and immediately thawed (one time), followed by three washes in 1× phosphate-buffered saline (PBS). Staining was carried out by incubating worms in 500 μl of BODIPY 493/503 (1 μg/ml) for 1 hour at room temperature, shaking in the dark. After incubation, worms were washed three times with M9 to remove BODIPY. Worms were then left overnight at 4°C, in shaking, in 1× PBS. Last, worms were imaged using a fluorescent microscope or sorted using COPASS BioSort as described in "Large-particle flow cytometry" section above..

### Q40 aggregation

Worms were bleached, and eggs were grown to day 1 of adulthood on HT115 bacteria. Worms were imaged at days 1 and 5 of adulthood and then imaged using fluorescent microscopy (see relevant section). Q40::YFP was expressed in neurons or intestine using promoters *rgef-1p* or *vha-6p*, respectively. For normalization of protein, standard Western blotting protocols were used. One hundred age-synchronized animals were hand-picked into an M9 solution and lysed in SDS-lysis buffer using 3× cycles of 100°C boiling for 10 min followed by flash-freezing in liquid nitrogen. The sample was then centrifuged at 10,000*g* for 5 min to pellet cell debris. The top 15 μl was pulled off and run on a 4 to 20% Criterion TGX Precast gel using a standard protocol, probed using an anti-GFP antibody and a fluorescent LI-COR secondary antibody, and imaged on a LICOR Odyssey M similar to filter trap assay below.

For intestinal polyQ imaging, osmotic stress was applied to worms at day 1 of adulthood by moving animals on to a plate with 500 mM NaCl and imaged after 4 hours of incubation. Three biological replicates were done.

### Thrashing assay

Thrashing of worms was measured using the WormLab (MBF Bioscience) system. Worms were grown to day 1 of adulthood, washed off plates using M9 buffer, and then allowed to settle by gravity in an Eppendorf tube, washed once, and then moved to an empty 6-mm plate (without NGM). Worms were video-recorded, in three biological replicates, and their thrashing (body waves) was measured using WormLab 2.0 software. The size of each worm (length and width output from WormLab) to gate for worm events and thrashing were averaged across the gated population.

### Chemotaxis assay

Animals were synchronized by bleaching, and assay was conducted at day 1 of adulthood as previously described. Briefly, animals were raised on EV bacteria until day 1 of adulthood and washed three times using M9 buffer by gravity settling. Chemotaxis assay plates were prepared: 100 mm NGM plates, with 1 μl of attractant (benzaldehyde, 1:200; diacetyl, 1:1000) and 1 μl of diluent (EtOH) on opposite sides, with 1 μl of sodium azide in the same location, to immobilize worms once reaching their target. In equal distance, 30 to 50 worms were dispended and scored after 60 min. Chemotaxis index was calculated by *I* = (# worms at attractant at 60 min − # worms at diluent at 60 min) / total number of worms. Assays were repeated at least three times, and significance was assessed by one-way analysis of variance (ANOVA) with Tukey’s multiple comparisons test.

### Filter trap retardation assay

Worms grown to day 1 of adulthood were washed off the plate using M9, harvested, and flash-frozen in liquid nitrogen. Then, animals were suspended in lysis buffer [100 mM Hepes (pH 7.4), 300 mM NaCl, 2 mM EDTA, and 2% Triton X-100, with EDTA-free protease inhibitor cocktail (Roche)]. Next, animals were homogenized using the Precellys Tissue Homogenizer, with glass and zirconium beads (2 mm). Lysates were centrifuged (8000*g* for 5 min at 4°C), supernatant was removed, and protein was quantified using a BCA Protein Assay kit (Thermo Fisher Scientific, 23225). Protein samples were applied on to cellulose acetate membrane with 0.22-mm pore size (Schlechtes + Schule) and assembled in vacuum slot blotter (Bio-Dot, Bio-Rad). Membrane was washed five times with 0.2% SDS on the blotter and subjected to antibody incubation for detecting aggregated protein retained on the membrane. Membranes were incubated with anti-GFP antibody (1:1000 dilution in LI-COR blocking buffer) overnight in a cold room. Membrane was washed three times with tris-buffered saline + tween (TBST) and then incubated with secondary antibody (1:10,000 dilution in LI-COR blocking buffer). Membranes were washed three times with TBST and imaged using the Odyssey M Imaging System (LI-COR) to visualize the protein bands. Bands were then quantified using Fiji.
