# Supplementary material for: Glial-derived mitochondrial signals affect neuronal proteostasis and aging
Source: Sci Adv. 2023 Oct 13;9(41):eadi1411. doi: 10.1126/sciadv.adi1411 (PMC10575585; doi:10.1126/sciadv.adi1411)
Supplement: Supplementary file 1 — Figs. S1 to S6 Legends for tables S1 to S4 [file sciadv.adi1411_sm.pdf]

Science Advances
AAAS

Supplementary Materials for

Glial-derived mitochondrial signals affect neuronal proteostasis and aging

Raz Bar-Ziv *et al.*

Corresponding author: Andrew Dillin, dillin@berkeley.edu

Sci. Adv. **9**, eadi1411 (2023)
DOI: 10.1126/sciadv.adi1411

This PDF file includes:

Figs. S1 to S6
Legends for tables S1 to S4

Other Supplementary Material for this manuscript includes the following:

Tables S1 to S4

Supplementary Materials

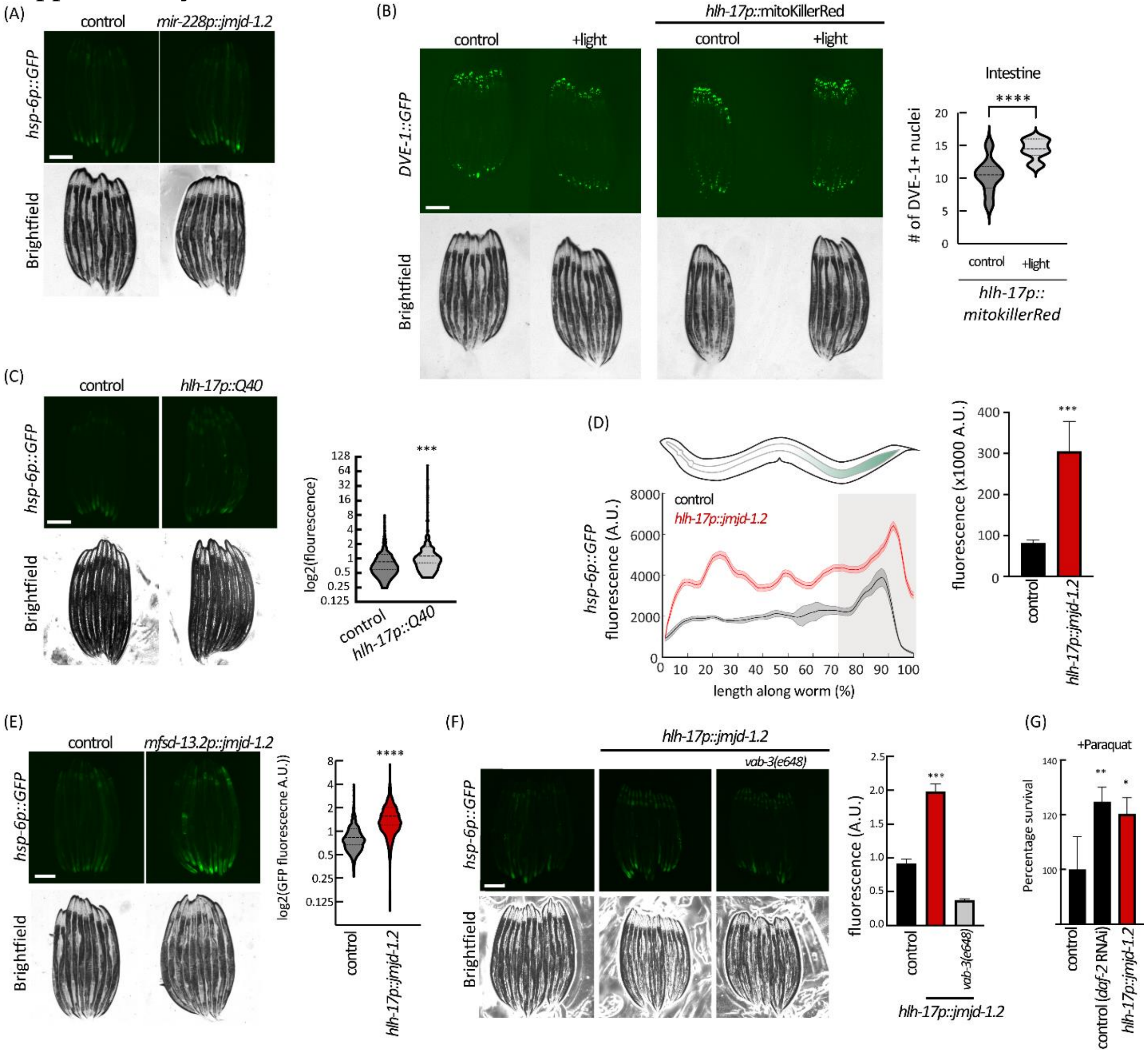

Figure S1: Activation of *jmjd-1.2* in glia induces UPR^{MT} in the periphery.

(A) Representative fluorescent micrograph of UPR^{MT} reporter *hsp-6p::GFP*, for worms expressing *jmjd-1.2* in most glial cells, under the *mir-228* promoter (*18*). (B) Same as S1A, for worms over-expressing mitochondrially targeted KillerRed construct, under the regulation of the *hlh-17* promoter, and the translational reporter DVE-1::GFP (left, see Figure 2A). L4 worms were irradiated with 1 min of light (543 nm excitation filter), and imaged after 24 hours, and quantified (right). unpaired Student's t test, ****P < 0.0001. (C) Same as S1A, for worms carrying an extrachromosomal array of the polyglutamine tract Q40 under the *hlh-17* promoter. (D) Worms over-expressing *jmjd-1.2* under *hlh-17p* were analyzed using a biosorter, and their spatial profiles aligned (left, see methods). The 30% most posterior part of the worm was defined as the posterior intestine, and the integral of the signal over the region was calculated (right). Unpaired Student's t test, ***P < 0.001. (E) Same as S1A, for worms over-expressing *jmjd-1.2* from a promoter identified as specific for CEPsh glia (*23*), *mfsd-13.2*p (C27H5.4) (left), and its quantification using biosorter (right). unpaired Student's t test, ****P < 0.0001. (F) As figure S1A, for *hlh-17p::jmjd-*

1.2 worms in combination with a mutation in the CEPsh glia lineage mutation in *vab-3* (*24*), and its quantification using biosorter (right). (G) The relative survival rate of worms (as percentage) exposed to paraquat was calculated as follows: Survival plot of each strain of worms was plotted over time, and the area under the curve (AUC) of the survival plot was calculated. The average AUC of the N2 strain was used as the control group. The normalized AUC is then multiplied by 100 to represent the percentage of survival. One-way analysis of variance (ANOVA) Tukey's multiple comparisons test, $^{**}P < 0.01$, $^{***}P < 0.001$, $^{****}P < 0.0001$.

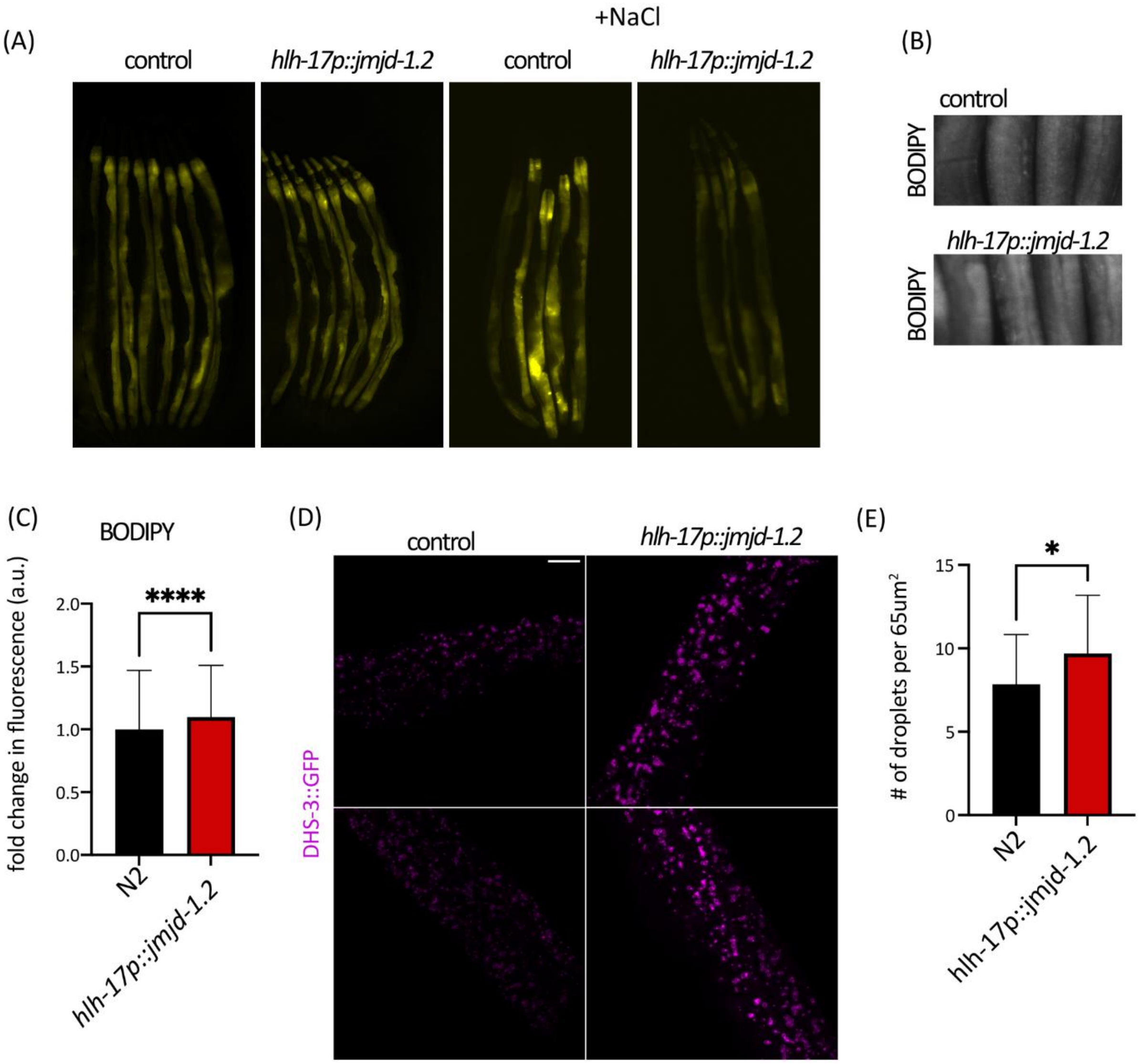

Figure S2: Glial activation of *jmjd-1.2* improves protein homeostasis in the periphery and increases lipid droplets.

(A) Representative fluorescent micrographs of Q40::YFP worms expressing in the intestine, after 4 hours in 400mM NaCl at D1 of adulthood. D1 adult animals normally do not show aggregation of Q40::YFP as it is mostly soluble under normal, healthy proteostatic environments. Hypertonic stress in the form of NaCl exposure can result in a 55-120% increase in aggregation (*31*). (B) BODIPY straining at D1 of adulthood and its quantification using worm biosorter in (C). (D) Imaging of DHS-3::GFP as in Figure 3 and number of lipid droplets were quantified using Fiji (E). Unpaired Student's t test, $*P < 0.05$, $****P < 0.0001$.

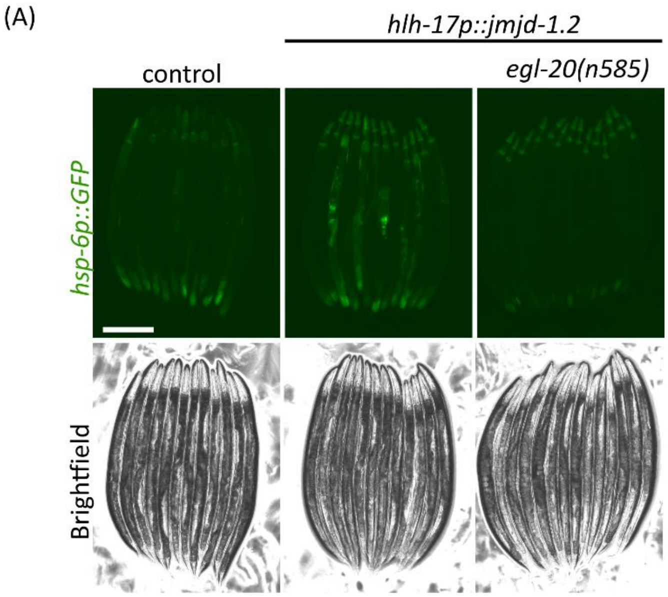

Figure S3: Cell non-autonomous activation by glial *jmjd-1.2* depends on WNT signaling. Representative fluorescent micrographs as in Figure 4, for worms mutated for the WNT ligand *egl-20* (*11*).

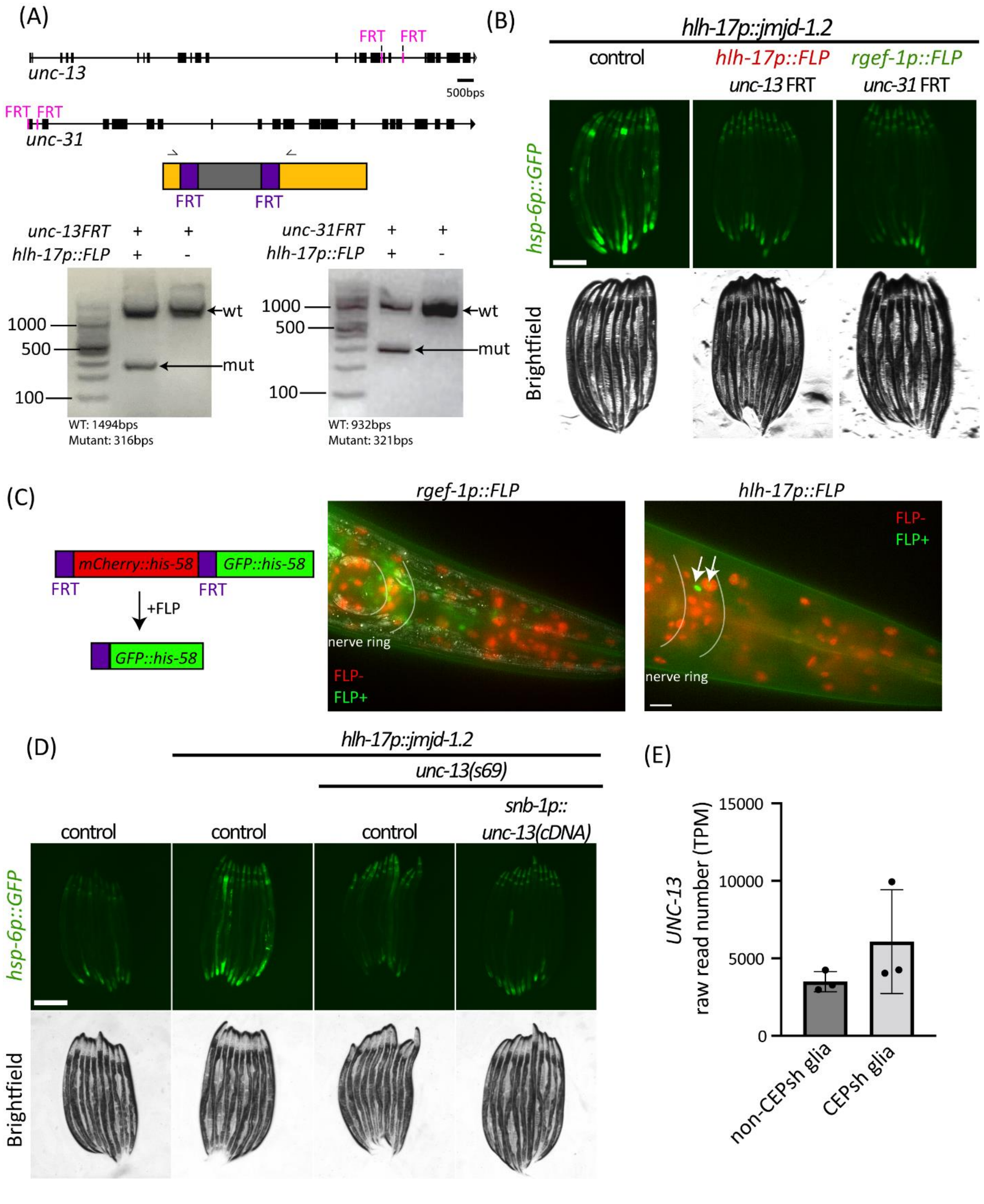

Figure S4: Cell non-autonomous activation by glial *jmjd-1.2* depends on WNT signaling. (A) Verification of cutting of newly generated FRT-strains for *unc-13* and *unc-31* (top scheme, FRT location in pink) was verified using PCR of whole-animal DNA extract, resulting in a shorter (cut) fragment when the FLP D5 was present. In addition, all strains were sequenced using Sanger sequencing to verify cutting. (B) Representative fluorescent micrographs as in

Figure 5. (C) Verification of the tissue-specificity of FLP alleles (*rgef-1p, hlh-17p*) used in this study was done using a reporter strain, which switches between red-nuclei (FLP negative) and green nuclei (FLP positive) (*37*). Arrows indicate CEPsh glia. (D) Rescue of *unc-13* in all neuronal cells could not rescue the activation of the UPR^{MT} in the intestine. (E) *unc-13* raw read counts in non-CEPsh glia and CEPsh glia.

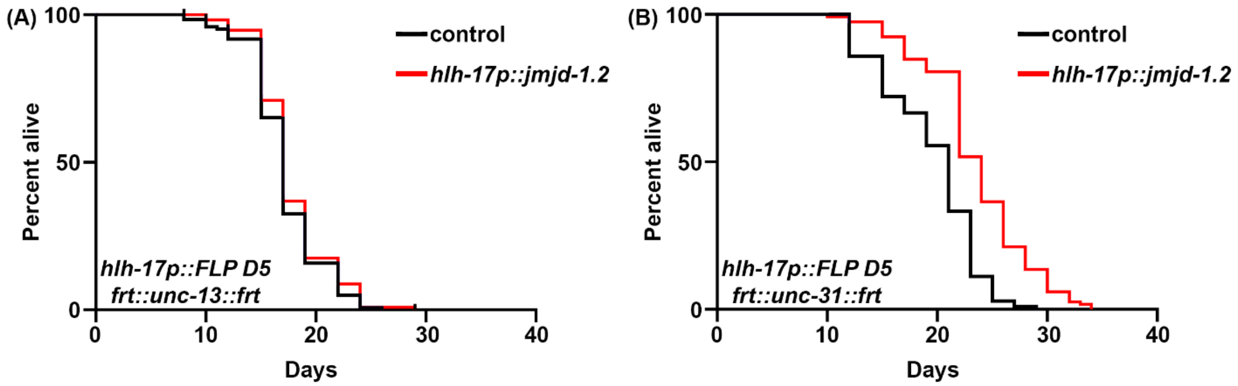

Figure S5: Lifespan extension of glial *jmjd-1.2* depends on glial SCVs.
(A) Survival of animals with CEPsh-glia specific knockout of *unc-13* in an otherwise wild-type background (black) or in animals with *jmjd-1.2* overexpression in CEPsh glia (red). P = 0.996.
(B) Survival of animals with CEPsh-glia specific knockout of *unc-31* in an otherwise wild-type background (black) or in animals with *jmjd-1.2* overexpression in CEPsh glia (red). P < 0.001.

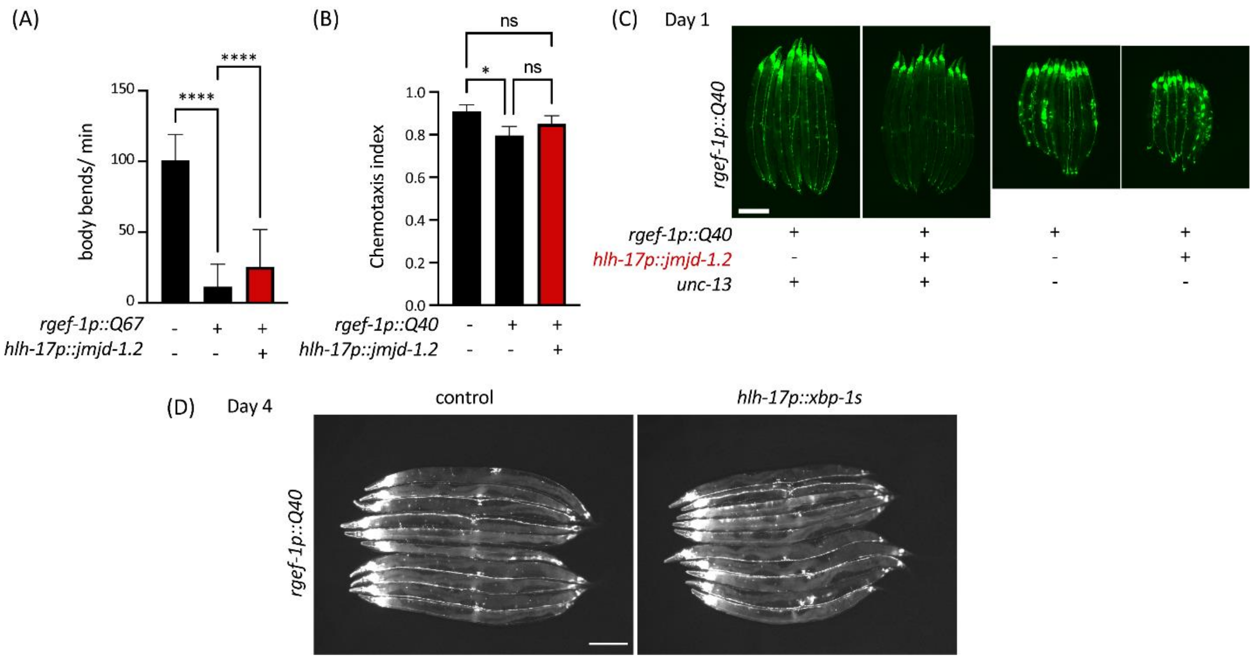

Figure S6: Glial *jmjd-1.2*, but not glial *xbp-1s*, rescues protein aggregation in neurons. (A) Thrashing of worms harboring the longer polyglutamine tract, Q67, in neuronal cells, as measured using the WormLab worm tracker (n>50). (B) Chemotaxis as in Figure 6B, towards diacetyl. (C) Same as in Figure 6D, for worms on D1 of adulthood. (D) As in Figure 6D, for worms expressing the ER-UPR transcription factor *xbp-1s* under the *hlh-17* promoter (*16*). One-way analysis of variance (ANOVA) Tukey's multiple comparisons test, n.s. non-significant, $*P < 0.05$, $****P < 0.0001$.

Table S1. DEGs in *hlh-17p::jmjd-1.2* animals.

Table S2. Enrichment of upregulated genes using gProfiler.

Table S3. GO enrichment for DEGs.

Table S4. Statistics for lifespan.
